# Medical Items

**Published:** 1917-10

**Authors:** 


### MEDICAL ITEMS

### THE CONTINUED USE OF COD LIVER OIL.

A marked disadvantage attaching to the long continued use of the ordinary emulsion of cod liver oil is the gastro-intestinal distress thereby occasioned. In many instances this distress makes is obligatory to discontinue the oil's use.

It was to obviate this bad feature of cod liver oil administration that Cord. Ext. OI. Morrhuae Comp. (Hagee) was perfected, and how well it has met the purpose for which it was intended is best shown by its wide popularity among the profession.

Cord. Ext. OI. Morrhuae Comp. (Hagee) offers every advantage of the plain cod liver oil, and owing to its palatability and easy assimitation it may be used indefinitely without causing gastro-intestinal disturbances.

### AT THE FRONT.

A great many physicians who have been in private practice are now or shortly will be in the service of the United State Government. Practically all of them are familiar with Antipholgistine, and a great number will continue to employ this preparation in their field work, as it is supplied by the Government on special requisition.

Since the beginning of the war tens of thousands of packages of Antiphlogistine have been used in the hospitals of France, at base hospitals in England, and in garrison and camp service in Canada, Australia and New Zealand.

The medical men who see service in France should rememb-er that Antiphlogistine obtained from The Denver Chemical Mrg. Co., 116 Rue de la Convention, Paris, is composed of the same C. P. ingredients and compounded according to the same formula as the American product.

